# INTERDISCIPLINARY MANAGEMENT OF OLDER ADULTS WITH HEARING LOSS: POLICY MATTERS

**DOI:** 10.1093/geroni/igad104.1469

**Published:** 2023-12-21

**Authors:** Michelle Arnold

**Affiliations:** University of South Florida, Tampa, Florida, United States

## Abstract

Hearing aids are an efficacious approach for treating hearing loss yet are underutilized in the US due to high out-of-pocket costs. Lack of hearing aid insurance coverage is one source of the breakdown in the interdisciplinary management of older adults with hearing loss, causing hesitation among primary care providers to refer patients to audiology for treatment. Recently, access and affordability of hearing aids for adults has gained traction, with several states mandating private insurance coverage of hearing aids. The purpose of this study was to understand state-level variability in private insurance hearing aid mandates and to quantify yearly averages of the share of privately insured adults aged <65. We conducted a longitudinal policy surveillance of state statutes for mandates through January 2023. Policy data were then combined with individual-level age, insurance, state, and year variables from American Community Survey and Medical Expenditure Panel Survey Insurance Component to estimate the share of privately-insured adults covered by a mandate from 2008-2022. We identified 25 states and 1 territory with effective private insurance hearing aid mandates. There was substantial variability in these mandates, including in exceptions, maximum age eligibility, allowable frequency of benefit use, and total coverage reimbursement amount. Between 2008-2022, we estimated that <10% of privately-insured adults lived in a state with coverage. We conclude that hearing aid mandates are covering a small but growing share of Americans. A federal mandate would be the fastest way to ensure access, but states can improve access by adopting exception-free mandates with limited utilization management.

